# Supplementary material for: Assessing suicidality during the SARS-CoV-2 pandemic: Lessons learned from adaptation and implementation of a telephone-based suicide risk assessment and response protocol in Malawi
Source: PLoS One. 2023 Mar 17;18(3):e0281711. doi: 10.1371/journal.pone.0281711 (PMC10022777; doi:10.1371/journal.pone.0281711)
Supplement: S1 File — (PDF) [file pone.0281711.s001.pdf]

Supplemental File 1: SHARP Study Safety Response Protocol for Phone Interviews

SHARP Study
Safety Response Protocol for Phone Interviews
Version 7 – 22.04.2020

USE THIS PROTOCOL FOR EACH PATIENT WHO REPORTS SUICIDAL THOUGHTS DURING A PHONE CONTACT

Suicidal Thoughts: Overview of Steps:

1. Complete Suicide Risk Assessment
2. Confirm patient's current location and who they are with
3. EITHER Complete Safety Plan for Active-High or Active-Emergent
4. OR Complete Safety Plan for Passive, Active-Low or Active-Moderate
5. Safety Follow-Up Schedule
6. Documentation of contacts

1. Suicide Risk Assessment (SRA)

Complete this protocol with all patients who reveal suicidal thinking in their response to question 9 on PHQ-9 (score >0 on ODK question C9).

A. PASSIVE VS. ACTIVE THOUGHTS

A.1. In the last two weeks, have you had any thoughts of hurting yourself in some way?

YES NO → ***STOP*. *Go to Section C.***

↓

ACTIVE THOUGHTS. Continue below:

A.2. How often have you had these thoughts in the last two weeks?

__ Once ___ A few times ___ Many times ___ Nearly every day

Continue to Section B.

B. DEGREE OF SEVERITY OF ACTIVE SUICIDAL THOUGHTS

		**Yes**	**No**	**Comments**
B.1.	In the past month, have you made any plans or considered a method that you might use to harm yourself. (If yes, ask "*Please be specific about these plans or methods you have considered.*")			
B.2.	Have you ever attempted to harm yourself? (If yes, ask, "*When was this? What happened*?")			

B.3.	There's a big difference between having a thought and acting on a thought. Do you think you might actually make an attempt to hurt yourself in the near future? (If yes, ask, "*Can you be specific about how you might do this*?")			
B.4.	In the past month have you told anyone that you were going to commit suicide, or threatened that you might do it? (If yes, ask, "*Who have you told and what have you said to them?*")			
B.5.	Do you think there is any risk that you might hurt yourself before you see your doctor the next time? (If yes, ask, "*What do you think you might do?"*)			

Continue on next page.

C. CURRENT SELF-HARM RISK

ASK THIS QUESTION FOR EVERY PARTICIPANT EVEN IF THEY SAID “NO” IN SECTION A.

C.1. Do you have any thoughts of hurting yourself right now?

YES NO

D. SUMMARY: DETERMINATION OF SUICIDE RISK LEVEL

Determine the participant’s suicide risk level using the table below.

<table>
<tr><th colspan="7">If the participant’s answers are…</th></tr>
<tr><td>A.1</td><td>Thoughts of hurting yourself in past two weeks</td><td>No</td><td>Yes</td><td>Yes</td><td>Yes</td><td rowspan="6">These answers don’t matter if C1 is yes</td></tr>
<tr><td>B.1</td><td>Made plans</td><td rowspan="5">Not asked</td><td>No</td><td rowspan="4">At least one yes</td><td rowspan="4">These answers don’t matter if B5 is yes</td></tr>
<tr><td>B.2</td><td>Ever attempted in past</td><td>No</td></tr>
<tr><td>B.3</td><td>Think you might hurt yourself in the near future</td><td>No</td></tr>
<tr><td>B.4</td><td>Told anyone you would hurt yourself</td><td>No</td></tr>
<tr><td>B.5</td><td>Might hurt yourself before seeing your doctor again</td><td>No</td><td>No</td><td>Yes</td></tr>
<tr><td>C.1</td><td>Thoughts of hurting yourself right now</td><td>No</td><td>No</td><td>No</td><td>No</td><td>Yes</td></tr>
<tr><td colspan="2">Then the Suicide Risk Level is…</td><td>Passive</td><td>Active-Low</td><td>Active-Moderate</td><td>Active-High</td><td>Active-Emergent</td></tr>
<tr><td colspan="2">In this row, mark the box corresponding to the participant’s answers and Suicide Risk Level</td><td></td><td></td><td></td><td></td><td></td></tr>
</table>

2. Confirm Patient’s Current Location and Who Else is There

A. Where are you right now?

____

B. Is anyone else there with you? If so, who?

____

IF SUICIDE RISK IS <u>ACTIVE-HIGH</u> OR <u>ACTIVE-EMERGENT</u>: CONTINUE TO SECTION 3.

IF SUICIDE RISK IS <u>PASSIVE</u>, <u>ACTIVE-LOW</u> OR <u>ACTIVE-MODERATE</u>: SKIP TO SECTION 4.

3. Safety Plan and Next Steps for Active-High or Active-Emergent

IF SUICIDE RISK IS <u>ACTIVE-HIGH</u> OR <u>ACTIVE-EMERGENT</u>: IMMEDIATE ACTION IS NEEDED FOLLOWING THE BELOW:

A. I am concerned about making sure you are safe. You said that ______________________ is nearby. ***Or if the participant said in Section 2 Question B that there is no one with them***: Is there someone nearby who I can talk to about your safety? Can you give me the phone number for that person or for someone else nearby that I can talk to about your safety? ***If the participant says that there is no one, continue to the next question.***

____

B. Can you please tell me the name of your nearest health post or health center? ___________________________________

C. If possible, can you please tell me the name and contact information of the HSA for your area? ____________________

____

D. Are you able to go to your nearest health facility so you can speak with a mental health professional? Is there someone with you who can help you get there? ***Note: This support person must be 18 years or older.***

____

E. Can I please speak with this person?

Note: If the participant refuses or says that no one is available in A or E, you must inform them that because you are concerned about their safety, you will contact the friend(s) or family member(s) whose number you have on the locator form. You must also inform them that if you are not able to make contact with a friend or family member to help, you will contact a health professional at the nearest health facility and ask them to contact the participant.

F. ***To the support person:*** PARTICIPANT has confidence in you, that is why I am talking to you, so please remember that this information we are discussing is confidential between you, PARTICIPANT, and any health professionals. PARTICIPANT has shared with me that they feel like they might harm themselves. This is a mental health issue that they need to speak to a health professional about. It is important for their safety that they speak to a mental health professional to be evaluated. Would you be able to help them go to your nearest health facility or call a mental health professional? I can give you contact information for the mental health professionals in this area. ***Send contact information for mental health professionals. Confirm that support person received them.***

Details about plan:

____

____

____

G. ***To the support person:*** I'd like to contact you tomorrow to hear what happened and how the participant is doing. When would be a good time to call? What number can I call to reach you?

____

H. May I speak to the participant again please?
To the participant: SUPPORT PERSON and I agreed that they would help you go to the nearest health facility or call a mental health professional. PROVIDE ANY OTHER DETAILS ABOUT PLAN. Do you have any questions?

I'd like to contact you tomorrow to hear what happened and how you are doing. Can I call this number? When would be a good time?

____
____

Thank the participant and end call. GO TO SECTION 5: SAFETY FOLLOW-UP.

NOTE: If you were not able to speak to a friend or family member to take charge of the participant, then you must call the SHARP NCD clinical coordinator at the participant's health facility.

- ***Inform the coordinator of the suicidal risk.***
- ***Inform them of the participant's phone number and location details, and any other phone numbers available on the locator form.***
- ***Document below your conversation with the coordinator and the action the coordinator has said they will take.***
- ***Follow Section 4: Safety Follow-Up and call the participant within an hour to see if the coordinator took the agreed-upon action. If they did not, call the coordinator again and document conversation again.***

____
____

4. Safety Plan and Next Steps for Passive, Active-Low or Active-Moderate

FOR ALL OTHER PARTICIPANTS (PASSIVE / ACTIVE-LOW / ACTIVE-MODERATE):

A. If you did start to feel like you might harm yourself, is there a friend or family member that you could tell about these feelings?

Who is this person?
____

Can you agree to tell this person if you do start to have thoughts about harming yourself?

____

If the participant cannot agree, go to Section 3 and follow the Active-High / Active-Emergent workflow instead.

B. Do you have access in your home to things you could use to harm yourself, like poisons, fertilizer, medications, or rope?

____
____

IF YES: Is there a friend or family member you can give these things to so that they are not in your house?

Name of person:
____

May I please talk to this person to confirm that you have given these items to them? [Document conversation with contact person):

____

____

If you are unable to confirm that the participant has handed over the potentially dangerous items, go to Section 3 and follow the Active-High / Active-Emergent workflow instead.

C. I'd like to make sure you can get help if you need it. Can you agree to go to your health facility or call a mental health specialist if you have thoughts of hurting yourself? ______________________________

If the participant cannot agree, go to Section 3 and follow the Active-High / Active-Emergent workflow instead.

D. I'm going to send you contact information for the mental health professionals in this area.
Confirm that the participant has received the contact information: ____________________

E. ***If Passive or Active-Low:*** I'd like to call you **in a week** just to check in. Is that all right? When is a good time?
If Active-Moderate: I'd like to call you **in 3 days** just to check in. Is that all right? When is a good time?

Date and time of scheduled call: ____

Confirm phone number to call: ____

F. In case I can't reach you, I'd like to make sure you're OK. If I can't reach you, is there a family member or a friend I can call to ask about you?

Family member or friend name and relation: ____

Phone number: ____

Thank the participant and end call. CONTINUE TO SECTION 5: SAFETY FOLLOW-UP.

5. Safety Follow-up

On follow-up phone call:

- Ask how the participant is doing
- If an action plan was agreed on during the last call (contact mental health professional; go to health facility), what is the update? Did the plan happen? If not, can it happen now?
- Ask Question 9 of the PHQ-9:
  - ***Since our last call, have you been bothered by thoughts that you would be better off dead or of hurting yourself in some way?***
    - If answer is NO: The participant has **No Suicidal Risk** at this time. Go to Summary of Follow-up Call Schedule below.

  - If answer is YES: Complete suicide risk assessment (Section 1) again. Then complete Section 2 and Section 3 based on those results. Then see Summary of Follow-up Call Schedule below.
- Agree on follow-up plan and next contact
- ***ALWAYS SAY:*** "If thoughts of hurting yourself return, please go to your nearest health facility or contact one of the mental health professionals whose numbers I gave you." ***Confirm that participant has these numbers from you.***
- Thank participant and end call
- ***If you are unable to reach the participant at the scheduled time: Call the support person instead, or other family members or friends listed on the locator form to enquire about the participant.***

Summary of follow-up call schedule:
- If participant started with **Passive** suicidal risk: Continue weekly follow-up.
  - If participant improves to **No** suicidal risk (answers "no" to question 9 of PHQ-9) at any point, then follow-up can end. As written above, be sure to confirm participant has mental health professionals contact information and will contact them if self-harm thoughts return.
  - If participant continues to have **Passive** suicidal risk, then after 3 weekly follow-ups, transition to ***ask the participant to contact you*** for the 4th weekly follow-up. If they do this successfully, then follow-up can end. As written above, be sure to confirm participant has mental health professionals contact information and will contact them if self-harm thoughts return.
- If participant started with **Active-Low** suicidal risk: Continue weekly follow-up.
  - If participant improves to **No** suicidal risk (answers "no" to question 9 of PHQ-9) at any point, then follow-up can end. As written above, be sure to confirm participant has mental health professionals contact information and will contact them if self-harm thoughts return.
  - If participant improves to **Passive** suicidal risk, then continue weekly follow-up. If participant continues to have **Passive** suicidal risk, then after 3 weekly follow-ups, transition to ***ask the participant to contact you*** for the 4th weekly follow-up. If they do this successfully, then follow-up can end. As written above, be sure to confirm participant has mental health professionals contact information and will contact them if self-harm thoughts return. If the participant remains at active-low for 3 follow-ups, the request that the participant initiates the 4th follow-up contact. If the participant successfully contacts the RA for the 4th weekly follow-up, follow-up can end.

- If participant started with **Active-Moderate** suicidal risk: Continue follow-up every 3 days.
  - If participant improves to **Active-Low** or **Passive** suicidal risk at any point, then follow-up can transition to weekly. If participant continues to have **Passive** suicidal risk, then after 3 weekly follow-ups, transition to ***ask the participant to contact you*** for the 4th weekly follow-up. If they do this successfully, then follow-up can end. As written above, be sure to confirm participant has mental health professionals contact information and will contact them if self-harm thoughts return.
  - If participant improves to **No** suicidal risk (answers "no" to question 9 of PHQ-9) ***at two consecutive weekly follow-ups***, then follow-up can end. As written above, be sure to confirm participant has mental health professionals contact information and will contact them if self-harm thoughts return.
  - If participant continues to have **Active-Moderate** suicidal risk after 3 contacts: Ask SHARP Coordinator for further direction.
- If participant started with **Active-High** or **Active-Emergent** suicidal risk: Continue follow-up every day.
  - If participant improves to **Active-Moderate** suicidal risk at any point, then follow-up can transition to every 3 days.
  - If participant improves to **Active-Low or Passive** suicidal risk at any point, then follow-up can transition to weekly. If participant continues to have **Passive** suicidal risk, then after 3 weekly

follow-ups, transition to ***ask the participant to contact you*** for the 4th weekly follow-up. If they do this successfully, then follow-up can end. As written above, be sure to confirm participant has mental health professionals contact information and will contact them if self-harm thoughts return.

- If participant improves to **No** suicidal risk (answers "no" to question 9 of PHQ-9) ***at two consecutive weekly follow-ups***, then follow-up can end. As written above, be sure to confirm participant has mental health professionals contact information and will contact them if self-harm thoughts return.
- If participant continues to have **Active-High** or **Active-Emergent** suicidal risk after 3 contacts: Ask SHARP Coordinator for further direction.

6. Summary of Follow-up Contacts

Date	Contact outcome	SRA Result	Action plan	Follow-up schedule	Follow-up date	Notes
Initial contact:		□ No risk<br>□ Passive<br>□ Active-Low<br>□ Active-Moderate<br>□ Active-High<br>□ Active-Emergent<br>□ Not assessed	□ NA<br>□ Plan:	□ None<br>□ 1 week<br>□ 3 days<br>□ 1 day<br>□ Other	□ NA<br>□ Date:	
Follow-up 1 date:	□ Called participant<br>□ Called support person<br>□ Participant called me<br>□ Support person called me<br>□ Tried to reach participant, not successful<br>□ Tried to reach support person, not successful	□ No risk<br>□ Passive<br>□ Active-Low<br>□ Active-Moderate<br>□ Active-High<br>□ Active-Emergent<br>□ Not assessed	□ NA<br>□ Plan:	□ None<br>□ 1 week<br>□ 3 days<br>□ 1 day<br>□ Other	□ NA<br>□ Date:	
Follow-up 2 date:	□ Called participant<br>□ Called support person<br>□ Participant called me<br>□ Support person called me<br>□ Tried to reach participant, not successful<br>□ Tried to reach support person, not successful	□ No risk<br>□ Passive<br>□ Active-Low<br>□ Active-Moderate<br>□ Active-High<br>□ Active-Emergent<br>□ Not assessed	□ NA<br>□ Plan:	□ None<br>□ 1 week<br>□ 3 days<br>□ 1 day<br>□ Other	□ NA<br>□ Date:	
Follow-up 3 date:	□ Called participant<br>□ Called support person<br>□ Participant called me<br>□ Support person called me<br>□ Tried to reach participant, not successful<br>□ Tried to reach support person, not successful	□ No risk<br>□ Passive<br>□ Active-Low<br>□ Active-Moderate<br>□ Active-High<br>□ Active-Emergent<br>□ Not assessed	□ NA<br>□ Plan:	□ None<br>□ 1 week<br>□ 3 days<br>□ 1 day<br>□ Other	□ NA<br>□ Date:	

Follow-up 4 date:	□ Called participant<br>□ Called support person<br>□ Participant called me<br>□ Support person called me<br>□ Tried to reach participant, not successful<br>□ Tried to reach support person, not successful	□ No risk<br>□ Passive<br>□ Active-Low<br>□ Active-Moderate<br>□ Active-High<br>□ Active-Emergent<br>□ Not assessed	□ NA<br>□ Plan:	□ None<br>□ 1 week<br>□ 3 days<br>□ 1 day<br>□ Other	□ NA<br>□ Date:	
Follow-up 5 date:	□ Called participant<br>□ Called support person<br>□ Participant called me<br>□ Support person called me<br>□ Tried to reach participant, not successful<br>□ Tried to reach support person, not successful	□ No risk<br>□ Passive<br>□ Active-Low<br>□ Active-Moderate<br>□ Active-High<br>□ Active-Emergent<br>□ Not assessed	□ NA<br>□ Plan:	□ None<br>□ 1 week<br>□ 3 days<br>□ 1 day<br>□ Other	□ NA<br>□ Date:	
Follow-up 6 date:	□ Called participant<br>□ Called support person<br>□ Participant called me<br>□ Support person called me<br>□ Tried to reach participant, not successful<br>□ Tried to reach support person, not successful	□ No risk<br>□ Passive<br>□ Active-Low<br>□ Active-Moderate<br>□ Active-High<br>□ Active-Emergent<br>□ Not assessed	□ NA<br>□ Plan:	□ None<br>□ 1 week<br>□ 3 days<br>□ 1 day<br>□ Other	□ NA<br>□ Date:	
Follow-up 7 date:	□ Called participant<br>□ Called support person<br>□ Participant called me<br>□ Support person called me<br>□ Tried to reach participant, not successful<br>□ Tried to reach support person, not successful	□ No risk<br>□ Passive<br>□ Active-Low<br>□ Active-Moderate<br>□ Active-High<br>□ Active-Emergent<br>□ Not assessed	□ NA<br>□ Plan:	□ None<br>□ 1 week<br>□ 3 days<br>□ 1 day<br>□ Other	□ NA<br>□ Date:	

Follow-up 8 date:	□ Called participant<br>□ Called support person<br>□ Participant called me<br>□ Support person called me<br>□ Tried to reach participant, not successful<br>□ Tried to reach support person, not successful	□ No risk<br>□ Passive<br>□ Active-Low<br>□ Active-Moderate<br>□ Active-High<br>□ Active-Emergent<br>□ Not assessed	□ NA<br>□ Plan:	□ None<br>□ 1 week<br>□ 3 days<br>□ 1 day<br>□ Other	□ NA<br>□ Date:	
Follow-up 9 date:	□ Called participant<br>□ Called support person<br>□ Participant called me<br>□ Support person called me<br>□ Tried to reach participant, not successful<br>□ Tried to reach support person, not successful	□ No risk<br>□ Passive<br>□ Active-Low<br>□ Active-Moderate<br>□ Active-High<br>□ Active-Emergent<br>□ Not assessed	□ NA<br>□ Plan:	□ None<br>□ 1 week<br>□ 3 days<br>□ 1 day<br>□ Other	□ NA<br>□ Date:	
