# Supplementary material for: Assessing suicidality during the SARS-CoV-2 pandemic: Lessons learned from adaptation and implementation of a telephone-based suicide risk assessment and response protocol in Malawi
Source: PLoS One. 2023 Mar 17;18(3):e0281711. doi: 10.1371/journal.pone.0281711 (PMC10022777; doi:10.1371/journal.pone.0281711)

Supplemental File 3: SHARP Safety Response Protocol for Phone Interviews Decision Tree for Passive and Active-Low Suicide Risk Follow-Up.

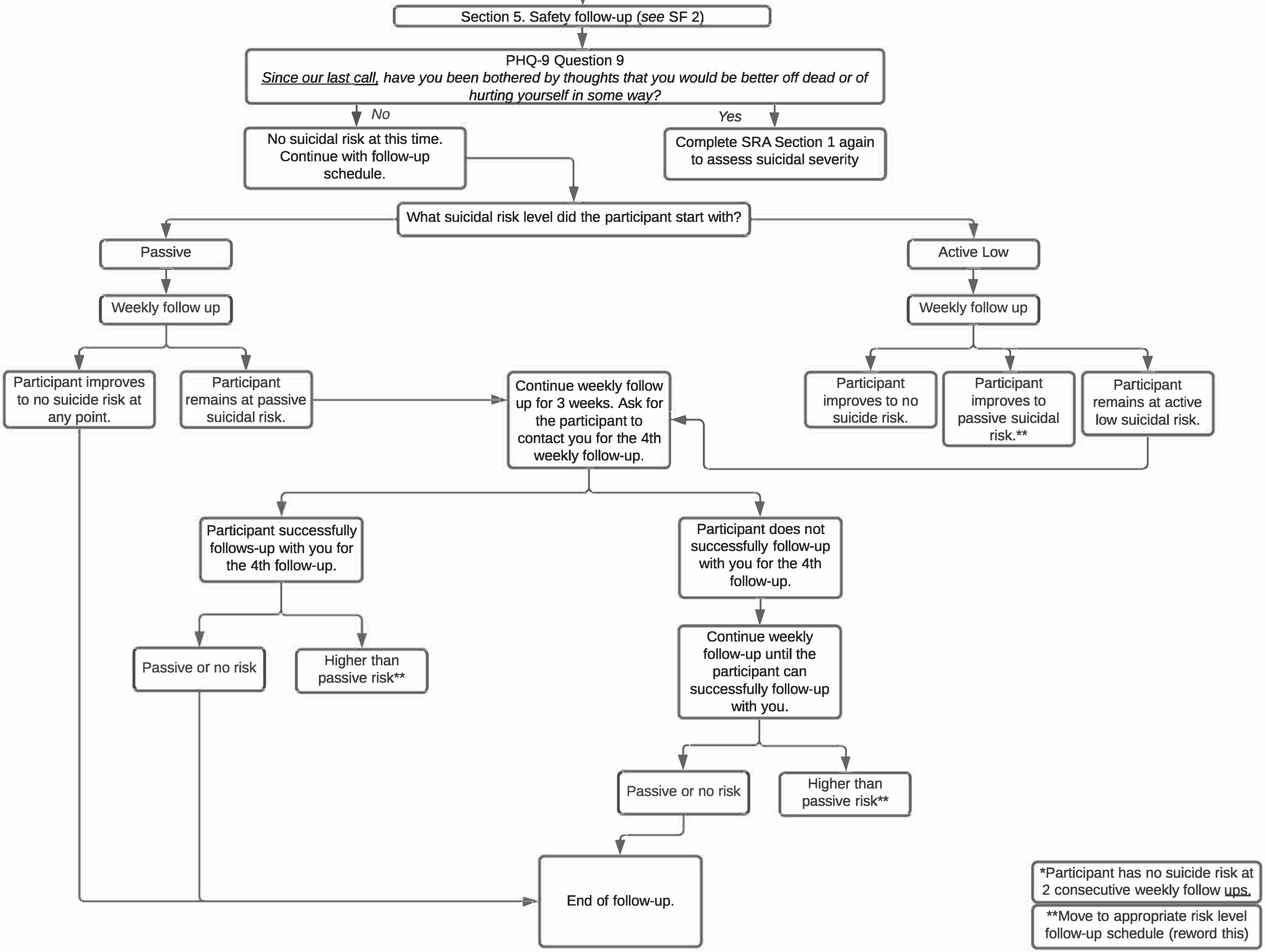

Supplement: S3 File — (PDF) [file pone.0281711.s003.pdf]
