# Supplementary material for: Assessing suicidality during the SARS-CoV-2 pandemic: Lessons learned from adaptation and implementation of a telephone-based suicide risk assessment and response protocol in Malawi
Source: PLoS One. 2023 Mar 17;18(3):e0281711. doi: 10.1371/journal.pone.0281711 (PMC10022777; doi:10.1371/journal.pone.0281711)
Supplement: Attachment — Submitted filename: Response_to_reviewers.pdf [file pone.0281711.s007.pdf]

November 11, 2022

PLOS One
RE: Manuscript Title: “Assessing suicidality during the SARS-CoV-2 pandemic: Lessons learned from adaptation and implementation of a telephone-based suicide risk assessment and response protocol in Malawi”, Manuscript Number: PONE-D-22-16562R1

Dear Reviewers 1 and 2,

Thank you very much for your time and thoughtful comments on our manuscript submitted to PLOS ONE, titled “Assessing suicidality during the SARS-CoV-2 pandemic: Lessons learned from adaptation and implementation of a telephone-based suicide risk assessment and response protocol in Malawi”. These comments are helpful in revising our manuscript. We have responded to your comments in line below, as well as in the manuscript through tracked changes. We hope that these changes adequately address your questions and comments.

Sincerely,

Kelsey R. Landrum

Reviewer #1: The SARS-CoV-2 pandemic led to the rapid transition of many research studies from in-person to telephone follow-up. It calls on efforts to develop and adapt protocols to fit this change. This paper describes the feasibility of development and implementation of a suicidality assessment protocol. It assessed its feasibility for assessing suicidality over telephone among patients who screen positive during the SRA, described the information collected and categorized, and described the primary challenges identified and lessons. This research belongs to a much needed type of studies to solve the urgent issues we face. The protocol / study is well described and the changeless and lessons are well discussed.

I just have a minor comment suggesting clarifying some numbers of the results—there were 689 patients meeting the criteria, but there are only 604 SI screens took place? There were 13 PHQ-9 positive, but only 12 received SRA?
Thank you for this comment and clarification. We have updated the data since this manuscript was submitted for review. The new manuscript addresses this discrepancy (as one participant had been receiving SRAs due to prevalent SI identified earlier in the parent study and not on a PHQ9 screening during the time period for the current analysis). The updated manuscript clarifies this and contains up-to-date data.

In Abstract, the abbreviate SI was first used without the full name spelled (line 59), and then was done on line 61.
Thank you for this comment. We had ensured that suicidal ideation is first spelled out, then abbreviated appropriately.

Reviewer #2: it's an interesting and topical important study to be conducted and applied to other setting in the future. Overall, a good manuscript. Few questions seeking for clarification:
1. Are there any expert validation being done? Could elaborate further in line 199?
Thank you for this question. Given the immediate need for a suicidality protocol during the pandemic, the guide was developed by subject matter experts and iteratively adapted. We have clarified this in the 'Protocol development section'.

2. Who provided the training- further elaborate in line 205
Research assistance were trained by subject matter experts in clinical psychiatry, health behavior, and epidemiology. We have clarified this in lines 208-210.

3. Would the RA has specific academic or research background requirement? Kindly also include that, if any.
All RA's had a minimum of a high school education level and were trained in SHARP study procedures and suicidality protocol procedures. We have clarified this in lines 208-209.

4. Was English the communication and main & only language used in the validation process with the participants?
Thank you for this clarification. The protocol was conducted in Chichewa or Chitumbuka, with responses translated into English (lines 212-215). We have ensured this is clarified in the 'Protocol development' section.

5. Line 169-175; there was a mixed of person interviews+ telephone being the data collection method. Would this present as a form of limitation? If yes- perhaps to include as limitation? If not- how do you mitigate the effect?
Thank you for this comment. We agree that this could be a limitation in that participant responses to interview questions may differ by mode of interview (in person versus via phone) and have added clarification about this potential form of bias in our discussion section. The study team, including a psychiatry study team member, conducted weekly reviews of all SRAs and patient follow-ups to ensure timely receipt of clinical care for all patients being followed up for SI. As we note, the phone protocol may be able to less accurately predict active-low compared to active-moderate suicide risk compared to the in-person assessment. In other words, as discussed in the discussion, the protocol tends to perform conservatively and classify patients as having a higher severity level when it is unclear if patients are a true low severity level. The literature has not measured the extent of this limitation, as most SRAs are conducted in person by clinical staff, but we have added more discussion of this potential limitation.[1–7]

REFERENCES

1. Harbauer, G., Ring, M., Schuetz, C., Andreae, A. & Haas, S. Suicidality assessment with PRISM-S—Simple, fast, and visual: A brief nonverbal method to assess suicidality in adolescent and adult patients. *Crisis: The Journal of Crisis Intervention and Suicide Prevention* **34**, 131–136 (2013).
2. Viguera, A. C. *et al.* Comparison of Electronic Screening for Suicidal Risk With the Patient Health Questionnaire Item 9 and the Columbia Suicide Severity Rating Scale in an Outpatient Psychiatric Clinic. *Psychosomatics* **56**, 460–469 (2015).
3. Forkmann, T. *et al.* Assessing suicidality in real time: A psychometric evaluation of self-report items for the assessment of suicidal ideation and its proximal risk factors using ecological momentary assessments. *Journal of Abnormal Psychology* **127**, 758–769 (2018).
4. Ellis, T. E., Rufino, K. A. & Allen, J. G. A controlled comparison trial of the Collaborative Assessment and Management of Suicidality (CAMS) in an inpatient setting: Outcomes at discharge and six-month follow-up. *Psychiatry Research* **249**, 252–260 (2017).
5. Exbrayat, S. *et al.* Effect of telephone follow-up on repeated suicide attempt in patients discharged from an emergency psychiatry department: a controlled study. *BMC Psychiatry* **17**, (2017).
6. Garza, N. D. L. *et al.* The Concise Health Risk Tracking Self-Report (CHRT-SR) assessment of suicidality in depressed outpatients: A psychometric evaluation. *Depression and Anxiety* **36**, 313–320 (2019).
7. Herbeck Belnap, B. *et al.* Electronic protocol for suicide risk management in research participants. *Journal of Psychosomatic Research* **78**, 340–345 (2015).
